# Inhibition of the P2Y2 Receptor Promotes Facial Nerve Function by Enhancing Neuron Autophagy

**DOI:** 10.2174/011570159X349328250717113503

**Published:** 2025-08-08

**Authors:** Xianmin Song, Yingna Gao, Minhui Zhu, Hongliang Zheng, Wei Wang, Shicai Chen

**Affiliations:** 1 Department of Otolaryngology-Head & Neck Surgery, Changhai Hospital, The Second Military Medical University, Shanghai, 200433, China

**Keywords:** P2Y2R, autophagy, mTOR, facial nerve injury, nerve regeneration, signaling pathways

## Abstract

**Objective:**

Facial nerve injury induces autophagy and apoptosis in facial nerve nucleus motoneurons of the CNS, impairing nerve regeneration and functional recovery. The function of P2Y2R after facial nerve injury remains to be determined. This study hypothesizes that inhibiting P2Y2R may play a protective role in facial nerve injury by modulating the autophagy signaling pathway.

**Methods:**

An *in vivo* mouse model of facial nerve crush injury was utilized in this study. Mice received either a P2Y2R agonist or antagonist through intrathecal injections of 10 μL/daily for 4 weeks. This study measured facial nerve function, examined fibrogenesis, and analyzed expression of autophagy regulatory proteins. In an *in vitro* experiment, NSC34 cells were treated with a P2Y2R agonist or an antagonist, and changes in the levels of phosphorylated PI3K, Akt, and mTOR, as well as autophagy regulatory proteins determined.

**Results:**

Inhibition of P2Y2R significantly increased autophagy levels and enhanced facial nerve function. These protective outcomes were linked to the suppression of phosphorylated PI3K, Akt, and mTOR signaling pathways.

**Conclusion:**

The study suggests that P2Y2R inhibition may improve facial nerve function by improving autophagy, making it a promising therapeutic approach for treating facial nerve injury.

## 1 INTRODUCTION

Peripheral nerve injury is a common clinical condition that often results in axonal degeneration, the loss of neurons, and muscle denervation [1-3]. Studies revealed that there are several molecular responses to the recovery of motor function following peripheral nerve damage. Degenerative processes, such as neuronal loss, demyelination, and impairments in cognitive function, start after nerve injuries.

According to recent research, autophagy is one of the cellular processes altered following peripheral nerve injury [4, 5]. Axon injury has been shown to upregulate essential autophagy genes, including Ambra1, Atg5, Beclin 1, and LC3 in both CNS and PNS [6, 7]. Autophagy is an essential intracellular mechanism that maintains cellular viability and stability by recycling and breaking down damaged proteins and organelles [8]. Numerous studies indicate that autophagy plays a neuroprotective role in neurological injury, including spinal cord and brain injuries [9-11]. Therefore, activation of autophagy may contribute to the recovery and treatment of motor neurons (MNs) after nerve injury. The knockout of autophagy genes (Atg5 or Atg7) in the nervous system or a specific neuronal cell type can lead to axonal degeneration and neuronal death in mice [12-14].

Adenosine 5′-triphosphate (ATP) is a crucial neurotransmitter that regulates neurological functions in both the central and peripheral nervous systems. Several studies have revealed that under pathological conditions, neurons, astrocytes, and microglia release high concentrations of ATP, which acts as a neurotransmitter or neuromodulator of the P2Y receptors [15, 16]. P2Y2R, which is expressed at low levels in neurons, is upregulated under pathological conditions [15, 17, 18]. P2Y2R, a member of the purinergic P2Y receptor family, mediates a variety of physiological and pathological responses through multiple and divergent intracellular signaling pathways. The activation of P2Y2 and P2Y4 receptors has been proposed as a potential treatment for neurodegenerative diseases [19, 20].

The study found that inhibiting P2Y2R can enhance neuronal differentiation and angiogenesis post-spinal cord injury [21]. Research on human 1321 N1 astrocytoma cells expressing recombinant P2Y2R indicates its crucial role in survival and neuroprotective mechanisms under pathological conditions [13]. Nevertheless, there is no report on whether P2Y2R mediates angiogenesis, nerve repair, and regeneration in the facial nucleus after nerve injury. Therefore, in the present study, the effects of P2Y2R on functional recovery and tissue sparing in a mouse model of facial nerve injury were explored, and the potential mechanism was examined.

## 2 MATERIALS AND METHODS

### 2.1 Animals

All animal procedures were approved by the Institutional Animal Care and Use Committee of the Second Military Medical University and associated guidelines on the ethical use of animals. Adult male C57BL/6 mice (20-30 g) were purchased from Beijing Vital River Laboratory Animal Technology Co., Ltd (Shanghai, China. The mice were randomly divided into four groups: sham, FNI, FNI + P2Y2R agonist, and FNI + P2Y2R antagonist groups. Adult male C57BL/6 mice 6-8week, P2Y2R agonist group n=50; P2Y2R antagonist group n=50; FNI groupn=50; sham group n=10.

### 2.2 Facial Nerve Injury Model

Adult male C57BL/6 mice (20-30 g) were anesthetized by intraperitoneal injection of 4% pentobarbital (30 mg/kg). The right extracranial facial nerve main trunk was exposed and crushed at a site 5 mm proximal to its bifurcation using forceps three times (10 seconds each time) using an operating microscope. In contrast, sham-operated mice were exposed but not subjected to crushing. For the P2Y2R antagonist group, after the model was established, P2Y2R agonist (2-ThioUTP tetrasodium salt, tocris, 1mM) and antagonist (AR-C118925XX, tocris,1mM) were intrathecally injected at 10 μL/d daily for 4 weeks [22]. Mice were divided into five subgroups based on injury time points (0, 7, 14, 21, and 28 days post-injury) and administered daily intraperitoneal treatment until they were sacrificed.

The facial nerve function was evaluated and scored. Absence of eye blinking and closure scored 1; the presence of orbicular muscle contraction, without blinking reflex, scored 2; 50% of eye closure through blinking reflex scored 3; 75% of closure scored 4; The presence of complete eye closure and blinking reflex scored 5; The absence of movement and posterior position of the vibrissae scored 1; slight shivering and posterior position scored 2; greater shivering and posterior position, scored 3 and normal movement with posterior position, scored 4; symmetrical movement of the vibrissae, with anterior position, scored 5.

### 2.3 Hematoxylin and Eosin Staining

The animals were sacrificed, and the facial nerve nuclei were harvested and fixed with 4% polymerized formaldehyde. The tissues were then paraffin-embedded and cut into 5-μm slices. Hematoxylin and eosin (H&E) staining was performed to observe the morphological changes in the facial nerve nucleus.

### 2.4 Cell Culture

Neuroblastoma x spinal cord cells NSC-34 were purchased from ATCC. Cells were cultured at 37°C, 5% CO_2_ in supplemented medium containing 1:1 DMEM/F12 (Ham) with 10% fetal bovine serum (FBS), penicillin, and streptomycin. Cells were subcultured every 2 days. The cells were then collected for Western blots and immunocytochemistry.

### 2.5 Cell Viability Detection by CCK-8 Assay

Cell viability was evaluated by the CCK-8 assay. Briefly, cells (1.0 × 10^4^/well) were plated in a 96-well microplate (three wells per group) in Dulbecco's Modified Eagle's Medium (DMEM) containing 10% fetal bovine serum (FBS) and 1% antibiotic-antimycotic and then incubated for 24 hours at 37°C with 5% CO_2_. Cells were treated with AR-C 118925XX/2-ThioUTP tetrasodium salt, respectively, and cultured for 12 hours. Then the medium was replaced, and 10 μL of CCK-8 (Dojindo, Kumamoto, Japan) was added to each well. The cells were further incubated for 2 hours, and the absorbance of each well was measured using a Microplate Reader at a wavelength of 450 nm according to the manufacturer’s instructions.

Cell viability was calculated according to the following equation:

Cell viability (%) = [(A − C)/ (B − C)] × 100.

Where A was the absorbance of the cells incubated with different concentrations of ethanol, B was the absorbance of the cells without ethanol, and C was the absorbance of the blank medium.

### 2.6 Western Blot Analysis

Cells were lysed with RIPA containing proteinase and phosphatase inhibitors (Sigma). Protein concentration was determined using the BCA protein assay method, with bovine serum albumin (BSA) as a standard. Fifty micrograms of protein samples were loaded per lane, separated by SDS-PAGE (10% polyacrylamide gels), and then electrotransferred onto nitrocellulose membranes. The membranes were blocked with 10% non-fat milk in Tris-buffered saline for 1 hour and incubated overnight at 4°C with P2Y2R (1:1000, Alomone), LC3(1:1000, Abclone), p62(1:1000, Abcam), p-PI-3K (1:1000, CST), PI-3k (1:1000, CST), AKT, pAKT (ser473), mTOR, pmTOR, and GAPDH (1:1000, Beyotime) antibodies diluted in 1% BSA in PBS. The membranes were then incubated with alkaline phosphatase–conjugated goat anti-rabbit IgG (Sigma) or goat anti-mouse IgG (Sigma) diluted 1:5000 in 2% BSA in PBS for 1 hour at room temperature. The Western blot results were visualized by Chemiluminescent HRP Substrate (Millipore, WBKLS0050, Bedford, MA, USA).

### 2.7 Transmission Electron Microscopy

To determine the number of autophagic vesicles in NSC34 cells, transmission electron microscopy was used. In brief, cells were fixed in fixative for TEM at 4°C for 2 hours, washed with 0.1 M PBS three times, and then post-fixed with 1% OsO4 in 0.1 M PBS (pH 7.4) for 2 hours at room temperature. Remove OsO4, dehydrate in a graded ethanol series, and embed in epoxy resin. Polymerization was performed at 60°C for 48 hours. Blocks were cut into ultrathin sections (70 nm) using a Reichert ultramicrotome. These sections were poststained with uranyl acetate in pure ethanol for 15 minutes and lead citrate, and observed under an electron microscope (JEOL, Japan).

### 2.8 Immunofluorescence

The cells were washed for 3-5 minutes in PBS, and then fixed in 4% (w/v) paraformaldehyde/PBS for 30 minutes at room temperature. The cells were preincubated in a blocking solution (10% normal bovine serum, 0.2% Triton X-100, 0.4% sodium azide in 0.01 mol/L PBS, pH 7.2) for 30 minutes followed by incubation with the primary antibodies: P2Y2R (1:1000), Alomone, rabbit polyclonal, APR-004; LC3 (1:400), Abclone, rabbit polyclonal, at room temperature overnight. Subsequently, the cells were incubated with Cy3-conjugated donkey anti-rabbit IgG (Jackson Immunoresearch) diluted 1:400 and FITC-conjugated donkey anti-mouse IgG (Jackson Immunoresearch) 1:200. All incubations were separated by 5-10 minute washes in PBS.

The average fluorescence was measured as the integrated density using ImageJ software for analysis, and at least three images were acquired per tissue section and cell treatment. Values are expressed as mean ± SEM.

### 2.9 Adenovirus (Ad)-mCherry-GFP-LC3-II Transfection and Confocal Microscopy

Cells were cultured in 24‐well plates at a density of 5 × 10^4^ cells/well and transfected with an adenovirus expressing the mCherry-GFP-LC3-II plasmid at a viral titer of 30 multiplicity of infection for 24 hours. Subsequently, the cells were treated with various agents at the indicated concentrations for another 24 hours. Then, the images of cells transfected with Ad‐GFP‐RFP‐LC3‐II were visualized with a Leica TCS SP5 laser scanning confocal microscope. Autophagy flux was measured by the color change of mCherry‐GFP. At least 30 cells of each group were analyzed.

### 2.10 Statistical Analysis

All the values are presented as the mean ± SEM and analyzed using GraphPad Prism 7.0 (GraphPad Software, Inc.). The statistical significance was determined by one-way analysis of variance (ANOVA) followed by a post-hoc Dunnett's test to compare the means of individual groups. The difference was considered significant when *p* < 0.05.

## 3 RESULTS

### 3.1 Facial Nerve Injury Enhances Autophagy and the Expression of P2Y2R

**Fig. (1):**
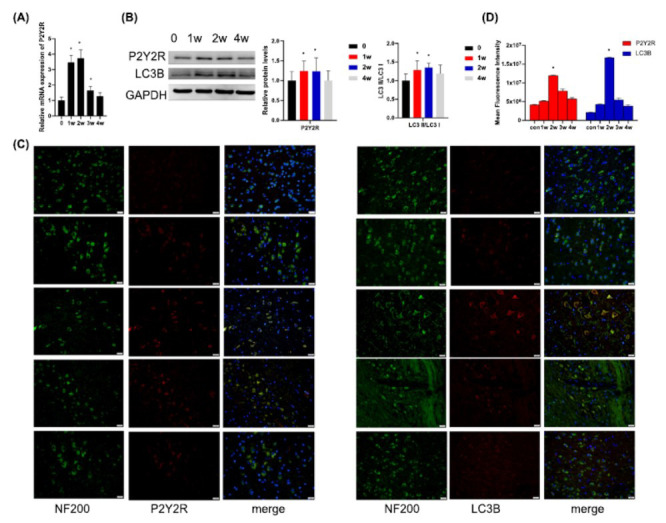
Expression of LC3 and P2Y2R in the facial nucleus after facial nerve injury. (**A**) The qRT-PCR analysis revealed altered expression levels of P2Y2R in the facial nucleus following facial nerve injury (n = 6, *p < 0.05, compared with control); (**B**) Western blots showing the expression of LC3 and P2Y2R in the facial nucleus after facial nerve injury; Bar graphs represent quantifications of the P2Y2R bands relative to GAPDH expression; *p < 0.05. Bar graphs represent quantifications of the LC3II/I relative to GAPDH expression; *p < 0.05; (**C**). Immunofluorescence staining was conducted to assess the expression of LC3 and P2Y2R in the facial nucleus following facial nerve injury, scale bar = 20 μm; (**D**). Bar graphs represent Mean Fluorescence Intensity, *p < 0.05.

The changes of autophagy in the facial motor nucleus after facial nerve injury are unclear. To investigate whether autophagy occurs after facial nerve injury and its relationship with P2Y2R, qPCR, Western blot, and immunofluorescence experiments were used to detect changes in autophagy markers (LC3) and P2Y2R expressions at weeks 1, 2, 3, and 4 after facial nerve injury. P2Y2R and LC3 expression levels were found to be significantly elevated, peaked at two weeks, and then gradually decreased (Figs. **1A**-**D**). This suggests the potential significance of P2Y2R and increased general autophagy activity in the facial nucleus following facial nerve injury.

### 3.2 Inhibition of P2Y2R Contributes to Autophagy

To determine if P2Y2R is associated with the induction of autophagy, P2Y2R agonist (2-ThioUTP tetrasodium salt) and antagonist (AR-C118925XX) were used in NSC34 cells to investigate the connection between the activation of P2Y2R and autophagy in motor neurons *in vitro*, first, cells were incubated with different agonist (2-ThioUTP tetrasodium salt) concentrations and antagonist (AR-C118925XX) for 24 hours. The CCK-8 assay results demonstrated that cell viability was decreased by P2Y2R agonist and increased by P2Y2R antagonist (Fig. **S1**).

**Fig. (2):**
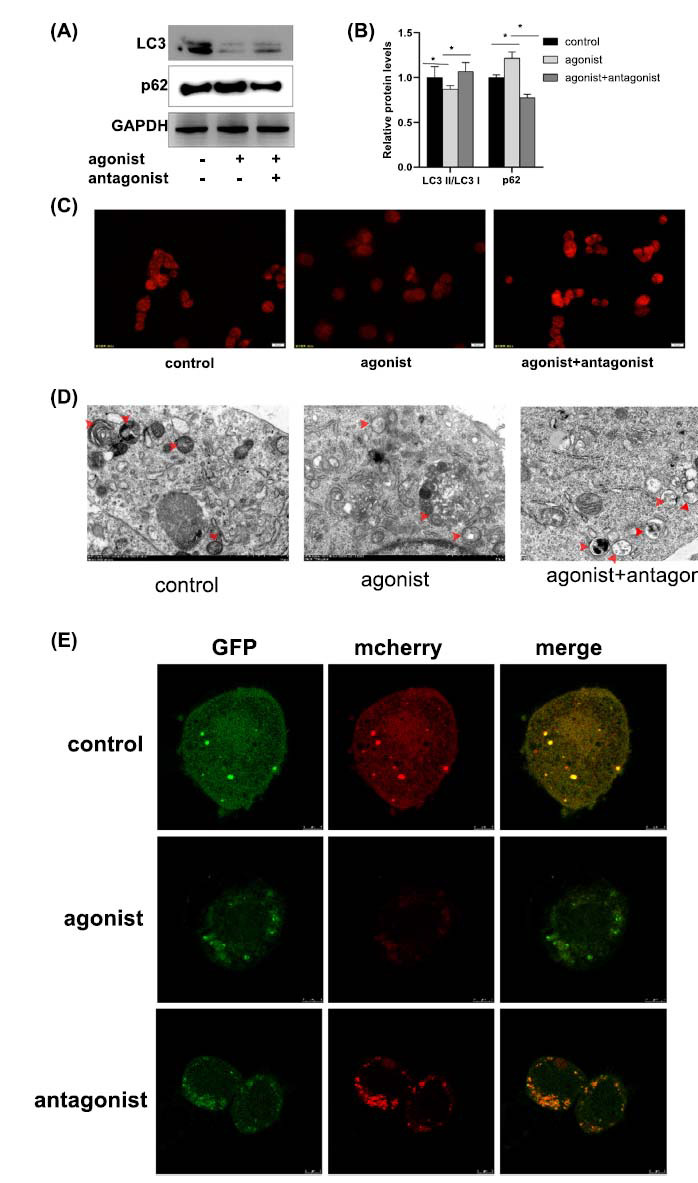
P2Y2R decreases autophagy in NSC34 cells. (**A**) Western blot analysis of LC3 and p62 expression in NSC34 cells after treatment with P2Y2R agonist and antagonist; (**B**) Bar graphs represent quantifications of the Western blotting bands relative to GAPDH expression; *p < 0.05; (**C**) Immunofluorescence staining of LC3 in NSC34 cells after treatment with P2Y2R agonist and antagonist, scale bar = 20 μm; (**D**). Transmission electron microscopy (TEM) analysis showing the formation of autophagosomes in NSC34 cells after treatment with P2Y2R agonist and antagonist, scale bar = 2 μm; (**E**). GFP-LC3 and mCherry-LC3 puncta were visualized using confocal microscopy, scale bar = 5 μm.

Western blot and immunofluorescence experiments were used to detect changes in LC3 expression in NSC-34 cells. The experimental results revealed that P2Y2R agonist increased p62 protein expression and decreased the ratio of LC3II/I, whereas the highly selective P2Y2 antagonist AR-C 118925XX enhanced autophagy (indicated by decreased p62 and increased ratio of LC3II/I) (Figs. **2A**-**C**). These results were also confirmed by transmission electron microscopy micrographs (Fig. **2D**). To confirm the effect of P2Y2R on autophagy, NSC-34 cells were transfected with Ad-mCherry-GFP-LC3b, and autophagosomes were then measured by confocal microscopy (Fig. **2E**). The results showed a small number of red and yellow spots in the control group. There was a significant increase in red and yellow spots in the P2Y2 antagonist group. These experimental results demonstrated that P2Y2R inhibition contributes to autophagy.

### 3.3 P2Y2R Regulates Autophagy *via* PI3K-Akt- mTOR Signaling Pathway

To investigate the potential mechanisms involved in P2Y2R-mediated autophagy in motor neurons, western blot analysis was used to measure the activation of PI3K, AKT, and mTOR, which are associated with autophagy or apoptosis.

**Fig. (3):**
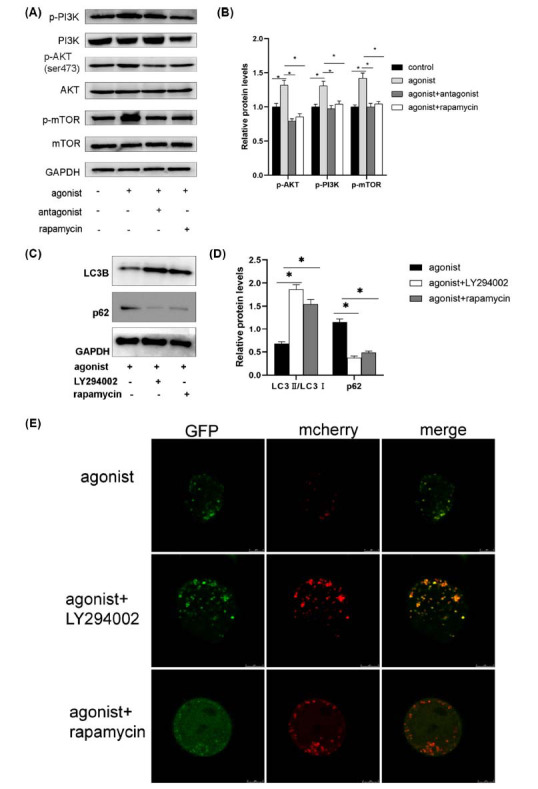
P2Y2R regulates autophagy *via* PI3K-Akt-mTOR signaling pathway. (**A**) Analysis of PI3K-Akt-mTOR expression in NSC34 cells following treatment with a P2Y2R agonist and antagonist was conducted using Western blotting techniques. (**B**) Bar graphs depict the quantification of Western blot bands concerning GAPDH expression, *p < 0.05; (**C**) Analysis *via* Western blot was conducted to assess the expression levels of LC3 and p62 in NSC34 cells post-treatment with a P2Y2R agonist and antagonist.; (**D**) Bar graphs illustrate the measurements of Western blot bands about GAPDH expression; *p < 0.05; (**E**) confocal microscopy was employed to visualize GFP-LC3 and mCherry-LC3 puncta, scale bar = 5 μm.

This study measured key signal proteins using Western blot analysis. As shown in the figure, treatment with a P2Y2R agonist caused an observable increase in p-PI3K, p-AKT, and p-mTOR. In contrast, the levels of p-mTOR (Figs. **3A**, **B**) were decreased in the P2Y2R antagonist group, indicating that PI3K-Akt-mTOR may be an important pathway in mediating the effects of P2Y2R. Furthermore, to determine the role of the PI3K-Akt-mTOR pathway in P2Y2R-mediated autophagy, LY294002 and rapamycin were used to determine the effect of P2Y2R on autophagy. LY294002 and rapamycin increased cell autophagy by reducing the LC3II/I ratio, while increasing p62 level and p-mTOR/mTOR ratio (Figs. **3C**, **D**). It was found that both GFP-LC3 and mCherry-LC3 were significantly upregulated in NSC34 cells after treatment with LY294002 and rapamycin (Fig. **3E**). These results reveal that inhibition of the PI3K-Akt-mTOR reverses autophagy flux impairment induced by P2Y2R agonist in NSC-34 cells.

### 3.4 P2Y2R Inhibitor Enhances Autophagy and Promotes the Repair of Facial Nerve Function

**Fig. (4):**
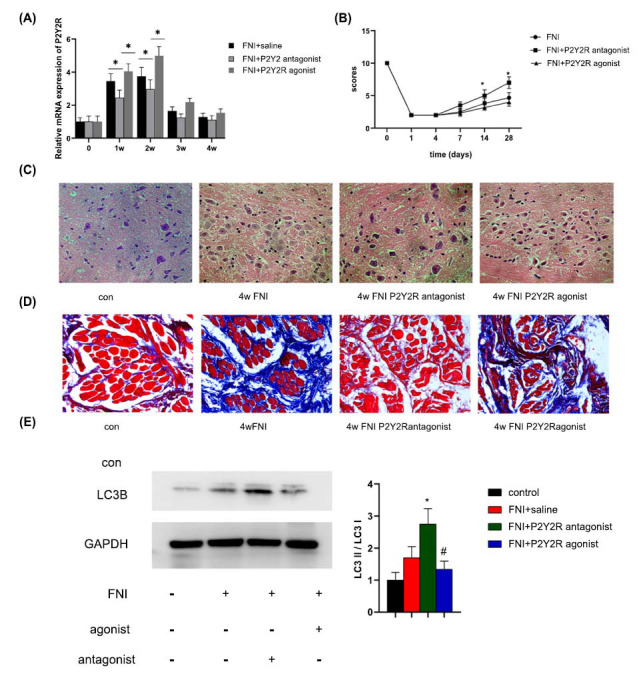
The functional and histological recovery of the injured nerve was assessed 4 weeks after treatment. (**A**) qRT-PCR analysis of the expression of P2Y2R in facial nucleus after treatment with P2Y2R agonist and antagonist (n = 6, *p < 0.05 compared with control); (**B**) Facial nerve function scoring was performed 0, 1, 4, 7, 14, and 28 days after surgery. The data were expressed as mean ± SEM (n = 6); *p < 0.05; (**C**) HE staining of facial nucleus; (**D**) Masson’s trichrome staining of facial muscles harvested 4-weeks; (**E**) Western blot analysis of LC3 expression in facial nucleus after treatment of P2Y2R agonist and antagonist after 4 weeks, Bar graphs represent quantifications of the Western blotting bands relative to GAPDH expression; **p < 0.05, compared with control, *p < 0.05, compared with antagonist group.

Following the establishment of the model, the P2Y2R agonist (2-thioUTP) or antagonist (Arc-118925XX) was administered intrathecally once a daily dose of 10 μL/d was established. At various timepoints (0, 1 w, 2 w, 3 w, and 4 w) following the injury, qPCR was used to identify the expression of P2Y2R. This study found that the expression of P2Y2R in the antagonist group was significantly decreased compared with the saline group and agonist group (Fig. **4A**, n = 6, *p < 0.05, compared with control). Neurological scores at 1, 7, 14, and 28 days were analyzed after injury, respectively. The analysis showed that, compared with the FNI + saline group, the FNI+P2Y2R antagonist group gained high neurological scores after 7 days and significantly higher scores at 28 days (Fig. **4B**, n = 6, *p < 0.05, compared with the control). Hematoxylin and Eosin (HE) staining of the facial nucleus showed that facial injury was greatly improved in the FNI+P2Y2R antagonist group (Fig. **4C**). Masson’s trichrome staining for fibrosis revealed a significant decrease in fibrosis in facial muscles at 4 weeks post-denervation compared with that in the control (Fig. **4D**). Western blot showed that LC3B was significantly increased in the FNI+P2Y2R antagonist group. These results confirm that inhibition of P2Y2R promotes the repair of facial nerve function (Fig. **4E**).

## 4 DISCUSSION

Peripheral nerve injuries result in partial or total loss of sensory, autonomic, and motor functions, impacting patients' quality of life. Functional recovery is often unsatisfactory, especially after severe injuries. Poor outcomes are attributed to long regenerative distances and slow axonal regeneration, leading to prolonged denervation and limiting the regenerative capacity of the distal nerve structure. However, many studies suggest that the pathogenic mechanisms and appropriate treatments for these pathological mechanisms have explored many new therapeutic methods for peripheral nerve injury. Nevertheless, finding new therapeutic targets is still imperative. This study investigated the role of P2Y2R in the facial motor nucleus after facial nerve injury in mice and found that P2Y2R is a potential pathogenic mediator by inhibiting autophagy, based on the following results. The results showed that the expression of P2Y2R was increased in the facial motor nucleus. P2Y2 deficiency could improve facial nerve function by activating autophagy signaling.

According to recent studies, extracellular ATP activates both membrane-anchored ionotropic P2X receptors and metabotropic P2Y receptors in various cell types. These receptors are associated with cell survival in regenerative conditions and modulate adult neuronal development and differentiation [23-26]. In the brain, P2Y receptors regulate neuron activity, neurovascular system function, and neuroinflammatory processes, promoting neuroprotective effects during inflammation.

Inhibitors of P2Y receptors inhibited the growth of neurites and the expression of neurofilament proteins and synaptic proteins induced by uridine and UTP [27]. Studies have shown that P2Y2 receptors play important roles in multiple functionalities [28-30].

P2Y2R/αv integrin interaction stimulates Rac and Rho, causing cytoskeletal rearrangements and regulating dendritic spines [31]. The use of conditioned medium from UTP-treated 1321N1 cells expressing P2Y2 receptors stimulates the outgrowth of neurites in PC12 cells [32]. Using the selective P2Y2R antagonist AR-C118925, satellite glial cells were completely inhibited from activating, exerting a significant anti-allodynic effect [33]. This study investigated whether P2Y2R plays a pathogenic or protective role in the facial nucleus during facial nerve injury development.

P2Y2R is important for the recruitment and activation of microglial cells, and the P2Y2R may regulate neuroprotective mechanisms through microglia-mediated clearance of Aβ in the TgCRND8 mouse model of Alzheimer's disease [34]. Chronic inflammation in the brain triggers the activation of the P2Y2R, which in turn recruits glial cells and phosphorylates the epidermal growth factor receptor (EGFR). It increases astrocyte migration and the expression of alpha(V)beta(3/5) integrins [35]. These data suggest that the P2Y2 receptor subtype also plays an important role in survival and neuroprotective mechanisms under pathological conditions. Nevertheless, there is no report on whether P2Y2R mediates angiogenesis, nerve repair, and regeneration after FNI.

Previous studies have reported that P2Y2Rs are expressed in neurons of the central and peripheral nervous systems, with a relatively low expression level [19]. P2Y2R is upregulated in inflamed or damaged tissues, including spinal cord injury [36] and brain injury [37], which suggests the possibility that the P2Y2R plays a protective role in the CNS. In this study, P2Y2R expression is elevated in mice with FNI, and inhibition of P2Y2R promotes nerve regeneration by increasing neuron autophagy. Autophagy plays an important role in the progression of neurodegenerative diseases. Numerous studies suggest that genetic therapies targeting the autophagic pathway may have a neuroprotective effect. CORT triggers PC12 cell injury and apoptosis through disrupting AMPK/mTOR‐mediated autophagy flux [38]. Increased miR-421-3p's binding to mTOR 3' UTR to reduce mTOR activity, increase autophagy, and decrease neuronal apoptosis in SCI mice [39]. Neurobehavioral defects and infarct volume were significantly reduced by normobaric oxygen treatment following ischemia/reperfusion through autophagy inhibition [40]. NeuroHeal promotes neuroprotection by regulating autophagy through the SIRT1 and AKT/PI3K axes [41]. This study found the activation of autophagic flux in the facial motor nucleus following facial nerve injury, indicating that autophagy plays a role in the potential progression of nerve regeneration.

This study showed that inhibition of P2Y2R markedly increased autophagy activity and enhanced the function of the facial nerve. A variety of signaling pathways are known to regulate autophagy, including the PI3K-Akt-mTOR pathway, the ERK and c-Jun N-terminal kinase (JNK) pathways, and AMPK pathways [42-44]. Studies have shown that after rat facial nerve injury, CXCL12 treatment improves facial nerve function and myelin regeneration by enhancing Schwann cell autophagy through the PI-3K-AKT-mTOR signaling pathway [45].

The results of the present study showed that activation of P2Y2R increased the levels of p-PI3K, p-AKT, and p-mTOR, while treatment with a P2Y2R antagonist decreased the levels of p-PI3K, p-AKT, and p-mTOR. The PI3K/AKT pathway plays a crucial role in cell physiology, participating in neuronal cell survival [46, 47]. Extracellular ATP activates P2Y2R, increasing AKT phosphorylation, which promotes inhibitory ULK-1 phosphorylation, and downregulates autophagy by inhibiting the Beclin-1 and ATG14 complex, which is necessary for phagophore formation [48]. According to this research, P2Y2R signaling triggers the PI3K/AKT signaling pathway, which inhibits the autophagy pathway and is thus detrimental to the pathophysiology of facial nerve injury. Changes in autophagosomes induced by P2Y2R activation were found to be reversible by pharmacological inhibition of the PI3K/AKT signaling pathway. This suggests that the PI3K/AKT/mTOR signaling pathway is involved in mediating the effects of P2Y2R.

In summary, this study found that autophagic flux decreased in mice following facial nerve injury, highlighting the crucial role of autophagy in neuroprotection. P2Y2R antagonist therapy successfully increased MN survival by triggering autophagy. P2Y2R antagonist treatment effectively promoted the survival rate of MNs by activating autophagy. Studies *in vitro* suggest that P2Y2R is involved in regulating autophagy through the PI3K/AKT/mTOR signaling pathway. The study emphasized the crucial role of P2Y2R in preventing and treating facial nerve injuries.

## CONCLUSION

This study demonstrates that in a mouse model of facial nerve injury, pharmacological inhibition of P2Y2R has neuroprotective effects by increasing autophagy and inhibiting apoptosis in motoneurons in the facial nucleus. The findings showed that both *in vivo* and *in vitro*, P2Y2R antagonists dramatically increase autophagy activity, decrease fibrogenesis, and improve facial nerve functional recovery. These advantages are mediated mechanistically by blocking the PI3K/Akt/mTOR signaling pathway, which is a crucial regulator of autophagy suppression.

## STUDY LIMITATIONS

A limitation of the current study regarding the role of P2Y2R is its reliance on the P2Y2R antagonist AR-C118925XX. Although this antagonist is a potent, selective, competitive, and reversible P2Y2 receptor antagonist, further validation using an alternative P2Y2R antagonist or P2Y2R gene silencing is necessary to confirm these findings. Additionally, the study relied on the P2Y2R agonist 2-ThioUTP tetrasodium salt, which acts not only on P2Y2 but also on P2Y4 and P2Y6 receptors, potentially complicating the interpretation of the results.

## Data Availability

The data and supportive information are available within the article.

## LIST OF ABBREVIATIONS

ATP: Adenosine 5′-triphosphate
BSA: Bovine Serum Albumin
EGFR: Epidermal Growth Factor Receptor
MNs: Motor Neurons
