# Supplementary material for: Inhibition of the P2Y2 Receptor Promotes Facial Nerve Function by Enhancing Neuron Autophagy
Source: Curr Neuropharmacol. 2025 Aug 8;24(2):230–40. doi: 10.2174/011570159X349328250717113503 (PMC13054728; doi:10.2174/011570159X349328250717113503)
Supplement: Supplementary file 1 [file CN-24-2-230_SD1.pdf]

Supplementary Material

Inhibition of the P2Y2 Receptor Promotes Facial Nerve Function by Enhancing Neuron Autophagy

Xianmin Song[1,#], Yingna Gao[1,#], Minhui Zhu[1], Hongliang Zheng[1], Wei Wang[1,*] and Shicai Chen[1,*]

[1]Department of Otolaryngology-Head & Neck Surgery, Changhai Hospital, The Second Military Medical University, Shanghai, 200433, China

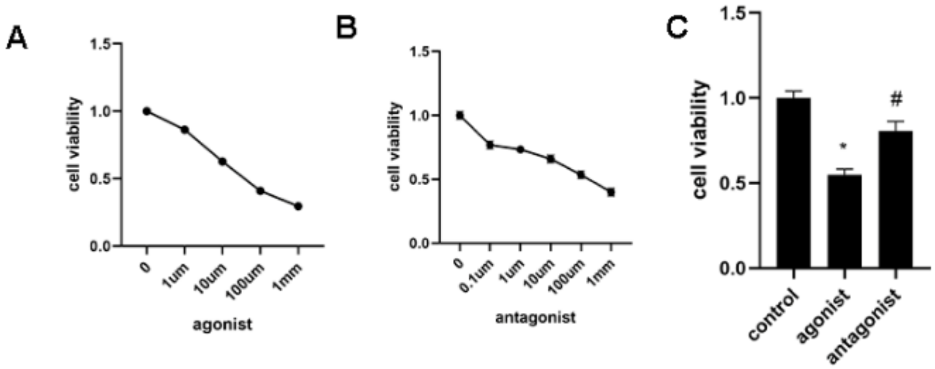

Fig. (S1). Cell viability in NSC34 cells. (**A**) Cells proliferation assay of NSC34cells after P2Y2R agonist treatment at 0,1,10,100 and 1000 μm/mL for 24 h by CCK-8 assay. (**B**) Cells proliferation assay of NSC34cells after P2Y2R antagonist treatment at 0,0.1,1,10,100 and 1000 μm/mL for 24 h by CCK-8 assay. (**C**) Cells proliferation assay of NSC34 cells after P2Y2R agonist(10μm) treatment at P2Y2R agonist +antagonist (1μm).

1875-6190/26 $65.00+.00 © 2026 Bentham Science Publishers
